# Full‐Season Injury Epidemiology in TeamGym—A Prospective Cohort Study Involving 474 Gymnasts

**DOI:** 10.1111/sms.70135

**Published:** 2025-09-14

**Authors:** Charlotte Anker‐Petersen, Mikkel Bek Clausen, Birgit Juul‐Kristensen, Per Hölmich, Carsten Bogh Juhl, Kristian Thorborg

**Affiliations:** ^1^ Department of Orthopedic Surgery, Sports Orthopedic Research Center‐Copenhagen, Amager‐Hvidovre Hospital Copenhagen University Hospital Hvidovre Denmark; ^2^ Department of Midwifery, Physiotherapy, Occupational Therapy and Psychomotor Therapy, Faculty of Health University College Copenhagen Copenhagen Denmark; ^3^ Department of Sports Science and Clinical Biomechanics, Research Unit for Musculoskeletal Function and Physiotherapy University of Southern Denmark Odense Denmark; ^4^ Department of Physioterapy and Occupational Therapy, Herlev and Gentofte Hospital University of Copenhagen Copenhagen Denmark

**Keywords:** epidemiology, gymnastics, injury, pain, text messaging, youth sports

## Abstract

Team gymnastics (TeamGym), a sport primarily practiced by girls/women, has rapidly gained popularity among adolescents. Despite a high pain prevalence among gymnasts, comprehensive epidemiological studies on injuries are lacking. This study aimed to investigate injury incidence rates, both overall and by body region including information on time‐loss and non‐time‐loss injuries, type and severity, in competitive TeamGym gymnasts aged 10–30 years. During a 10‐month season (August 2021–June 2022), a cohort of 474 gymnasts (73% women) was prospectively followed through weekly text‐message queries on injuries, time‐loss (partial or full absence from training/competition due to an injury), and gymnastic training and competition exposure. Injuries were verified and categorized through standardized telephone interviews. An injury with reported time‐loss of ≥ 4 calendar weeks was classified as severe. Incidence rates were calculated using Generalized Poisson regression. Totally 1382 injuries were recorded, and the overall incidence rate was 14.7 injuries per 1000 h of exposure (95% CI: 13.3–16.3) with an incidence rate of 14.0 for women and 17.2 for men. The foot (24.5%), knee (22.0%), lower leg (10.4%), and lower back (10.4%) were the most frequently injured regions overall in both sexes, and > 60% of the injuries were due to overuse. Incidence rate for severe injuries was 0.5 (95% CI: 0.4–0.7), which predominantly included injuries to the foot/heel (32%), lower back (21%), and knee (19%). The injury incidence rate in TeamGym is high, with three‐quarters of injuries being non‐time‐loss, and nearly two‐thirds caused by overuse. Over half of all injuries affected the feet, knees, and lower back across both sexes.

## 1 Introduction

Team gymnastics (TeamGym) is a relatively new, rapidly growing discipline, primarily practiced by girls and women, that has gained considerable popularity among children and adolescents [1, 2]. In TeamGym, athletes compete in teams of 8–10 participants (boys/men, girls/women, and mixed teams) in an acrobatic floor routine, tumbling, and trampette, aiming to achieve the highest possible total score [1, 2]. Unlike artistic gymnastics, TeamGym routines are identical for both sexes but are very similar to the floor and vault routines seen in artistic gymnastics. In contrast to rhythmic gymnastics, TeamGym does not use handheld equipment. Instead, powerful acrobatic jumps are a key element of the floor routines.

In TeamGym, a small study (*n* = 42) has reported an injury incidence rate of 2.2 new injuries per 1000 h of gymnastics [3], which is comparable to artistic gymnastics, where the injury rate is reported to range from 0.5 to 3.7 per 1000 h [4, 5]. Most injuries (62%–70%) seem to occur in the lower extremities [3, 6, 7] with ankle/foot, knee, and back being the most affected regions [6, 7]. Additionally, 21% of reported injuries were due to overuse [3].

These epidemiological data on TeamGym rely on only three studies: two cross‐sectional [6, 7] and one prospective cohort study [3]. The prospective study only assessed injuries resulting in time‐loss [3], and highlights a significant gap in knowledge concerning both time‐loss and non‐time‐loss injuries, as well as their anatomical distribution. Furthermore, no prospective studies have collected injury data while also closely monitoring gymnastics exposure at the sub‐elite TeamGym level. A larger prospective study is therefore warranted to provide a comprehensive epidemiological overview of TeamGym injuries. Such a study should distinguish between time‐loss and non‐time‐loss injuries, and also examine injury etiology and type.

Hence, this study aimed to prospectively investigate injury incidence rates overall, and for each body region including information on time‐loss, non‐time‐loss, type, and severity, in competitive TeamGym gymnasts aged 10–30 years, during a complete season of 10 months, using telephone text messages and interviews.

## 2 Material and Methods

### 2.1 Study Design

During the Danish 2021/2022 TeamGym season (August–June), a 42‐week prospective cohort study was conducted, involving competitive gymnasts aged 10 to 30 years from the National Liga (elite level) and Danish Series (sub‐elite level). Data collection included questionnaires, weekly text messaging, and telephone interviews. Ethical approval was granted by the Ethics Committee of the Capital Region (H‐20024056). The study is reported according to the Strengthening the Reporting of OBservational Studies (STROBE) criteria [8].

### 2.2 Equity, Diversity and Inclusion Statement

Our study sample was designed to represent the national TeamGym gymnast population, encompassing diverse demographic and socioeconomic backgrounds. Furthermore, our multidisciplinary author team includes various genders, professions (such as physiotherapy and orthopedics), and academic ranks, from junior researchers to professors.

### 2.3 Participants

Participants were recruited from 71 teams in nine of the 47 eligible competitive gymnastics clubs in Denmark. The nine clubs were selected to represent the broader TeamGym population and were located in socioeconomically diverse areas within a 90‐min drive of the research center. Study information was shared via a short video on private social media, followed by written and oral information during training sessions in spring and late summer 2021. Exclusion criteria at inclusion were pregnancy and gymnasts with serious musculoskeletal conditions, such as chronic joint disorders, recent fractures, or ongoing rehabilitation from major injuries.

### 2.4 Data Collection

In spring, gymnasts received envelopes containing two consent forms (for the gymnast and guardian), a demographics questionnaire (including contact information), and a generalized joint hypermobility (GJH) questionnaire. These documents were returned to the team coaches in sealed envelopes and were collected by the primary investigator by July 1, 2021. Gymnasts recruited in August and September completed and returned the same documents immediately after attending information meetings.

The demographics questionnaire included information on age, weight, height, years of TeamGym experience, and preferred take‐off leg. The Five‐Part Questionnaire (5‐PQ) [9] was used for classification of GJH [10, 11]. A score of two or more positive answers in the 5‐PQ indicated the presence of GJH [9]. The 5‐PQ has demonstrated satisfactory diagnostic accuracy (sensitivity 70.9%–91%; specificity 75%–77.4%) compared with the Beighton score [12, 13], the gold standard for diagnosing GJH.

**FIGURE 1:**
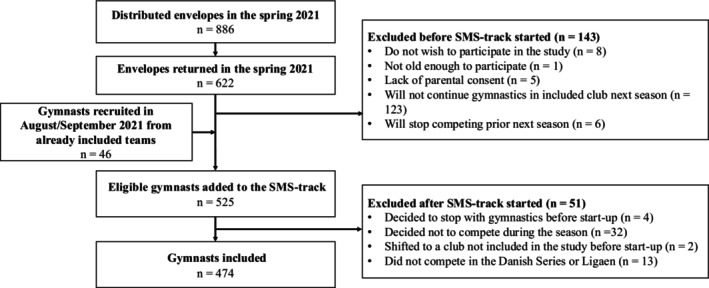
Flow chart describing the inclusion and flow of gymnasts throughout the study.

Participants recruited before July 1, 2021, began weekly text message questions after summer training ended, coinciding with regular training in August/September. Gymnasts recruited later started receiving text messages the Monday after inclusion (see Figure 1 for study flow).

Weekly, all participants answered four text message questions, see Table 1. The first question was sent Monday afternoon, with subsequent questions sent immediately after each response. If a participant did not respond, a reminder was sent Wednesday.

**TABLE 1:** Questions (Q) asked through the weekly text‐messaging system.

Q1	Did you experience any pain and/or discomfort during gymnastic training or competition last week? “No” (=0), “Yes” (=1), “Yes, because of the same problem as the week before” (=2)
Q2	Were you absent from training and/or competition last week? “No” (=0), “Yes, because of sickness” (=1), “Yes, because of pain/discomfort” (=2), “Yes, because of other reasons” (=3)
Q3	Have you been restricted in your training due to pain and/or discomfort last week? “No” (=0), “Yes” (=1), “Yes, because of the same problem as before” (=2)
Q4	How many hours and minutes have you been training and competing in gymnastics the last week?

To validate and gather more information about injuries, participants reporting gymnastic‐related pain or discomfort were contacted for a standardized 5‐min telephone interview (Appendix A). The interviews, conducted by two trained bachelor students under the guidance of the primary investigator, aimed to classify complaints based on predefined criteria described below. If participants could not be reached within 3 weeks, follow‐up attempts were made for up to eight additional weeks by the primary investigator. Only clear responses were recorded; unclear responses or participants not reached were categorized as “non‐classified”

Reports initially stated as new injuries related to gymnastics (identified as pain or discomfort, Q1 = 1) but unconfirmed during interviews were manually corrected to “no injury”.

#### 2.4.1 Sample Size Considerations

To ensure our study represented the competitive TeamGym population in Denmark, we aimed to recruit at least 20% of the approximately 1524 gymnasts aged 10–30 years in the Danish National Liga and Danish Series, including clubs from diverse socio‐economic areas.

### 2.5 Patient and Public Involvement

The standardized telephone interview was developed based on semi‐structured interviews with current and former TeamGym gymnasts and physiotherapists specializing in gymnastics.

### 2.6 Injury Definitions and Exposure

An injury was defined as any new gymnastic‐related pain or discomfort reported by the gymnast (Q1 = 1) and verified in the subsequent telephone interview. The injury was considered resolved when no pain/discomfort, restriction, or time‐loss was reported for 1 week during which training and/or competition was recorded. If a new injury occurred while another was ongoing, the initial injury's duration was considered ended, and the new injury was followed until its termination.

Injuries were classified as time‐loss or non‐time‐loss following the International Olympic Committee (IOC) Consensus Statement [14]. Based on this guide, time‐loss injuries were defined as those that, at any point during the injury period, prevented gymnasts from returning to normal training or competition [14]. For all weeks during an injury's duration, any week with partial (i.e., the injury led to missing at least one training session during the week) or full absence due to injury (Q2 = 2) was recorded as a time‐loss week. Injuries with ≥ 1 time‐loss week were classified as time‐loss injuries. Non‐time‐loss injuries were defined as injuries in which no time‐loss occurred at any time during the duration of the injury.

Injuries were further categorized as acute (resulting from a specific identifiable event) or overuse (gradual onset). Acute injuries were sub‐classified as training‐ or competition‐related. Medical attention was categorized according to the gymnastic‐specific extension of the IOC 2020 consensus statement [15]. Injured regions were identified based on reports provided by the gymnasts during interviews. If multiple injuries occurred on different days within the same week, all were recorded; however, only the most painful injury, as reported by the gymnast, was tracked for duration. In cases involving trauma to multiple regions, all affected regions were recorded and documented; however, the severity of injuries in each region was not documented, as tracking individual severity across multiple regions was not feasible within the data collection method used. For the same reason, only the more painful side was recorded and monitored when bilateral overuse injuries occurred in the same region simultaneously. If the gymnast could not determine which side was more painful, this was noted during the interview, but the injury was still recorded as a single injury in that region.

Severity of time‐loss injuries was recorded in line with the IOC Consensus Statement on injury surveillance [14], as the number of calendar weeks with ≥ 1 day of reported time‐loss during the injury. Injuries with ≥ 4 time‐loss weeks were classified as severe, which is consistent with the IOC's suggested threshold of > 28 days. Weekly reports independently recorded pain/discomfort due to gymnastics (Q1), restrictions in training/competition due to an injury (Q3), and time‐loss (Q2). Total weeks absent due to illness (Q2) were also recorded. Injuries were classified as new or recurrent during the telephone interview. Recurrent injuries were defined as injuries of the same type and location as a previous fully resolved injury—involved injuries occurring before or during the data collection period.

Training and competition exposure was calculated based on responses to the fourth text message question (Q4).

### 2.7 Statistics

Descriptive statistics, including training and competition exposure, injuries, and injury incidence rates, are presented at group level and by gender. Missing exposure data were not imputed. Demographic characteristics, including GJH, are reported as proportions, percentages, means, and standard deviations (SD). Training and competition exposure is calculated as average weekly exposure over the 42‐week data collection period, regardless of absence due to illness, injury, or holidays.

Generalized Poisson regression was used to estimate overall injury incidence rates and 95% confidence intervals (CI). At the body regional level, Poisson regression was used. A sensitivity analysis assessed the need for Generalized Estimation Equations to address team and club clustering. All missing values for injury and exposure were excluded from these analyzes, as were injuries that were ongoing at the time of inclusion. However, exposure time from injuries that could not be classified was included in the Poisson regression analyzes. The proportion of time‐loss injuries in a specific body region was computed by dividing the number of injuries in each region by the total number of time‐loss injuries. Corresponding 95% CIs were calculated using a normal approximation to binomial CIs. The proportions of severe time‐loss injuries were also determined for each region.

Missing responses to weekly text messages were imputed based on predefined rules to determine whether an ongoing injury had resolved. (1) If the week before the missing data indicated no injury, and the first response after reported a new injury (Q1 = 1), the injury started in the week it was first reported and validated through the telephone interview. (2) If the week before the missing response(s) showed an injury (either ongoing or new), and the first available response afterward reported no pain, no restrictions, and training exposure, the injury was assumed to have resolved in the week immediately before the missing data. (3) If an injury was considered ongoing in the week prior to the missing response, and the first response after the missing data showed ongoing pain, absence, or restrictions due to the same problem, the injury was considered ongoing during the missing week(s). These rules were applied with a maximum of four missing consecutive weeks, as participants who were nonresponsive for four consecutive weeks were classified as dropouts. At study‐end or at dropout, the duration of all ongoing injuries was stopped. Data from withdrawn gymnasts were included until their dropout week.

STATA 18 (StataCorp, College Station, Texas, USA) was used for the statistical analyzes.

## 3 Results

All nine invited gymnastic clubs participated. Of the 886 distributed envelopes, 622 were returned (70%). Before weekly text‐message data collection began, 143 gymnasts (23%) were excluded primarily due to quitting gymnastics or moving to a boarding school in another part of the country. In August and September 2021, 46 additional gymnasts were recruited from the included teams, increasing the number of eligible participants to 525. After data collection started, 51 gymnasts were excluded, reducing the prospective registration to 474 (90%) (see Figure 1). During the data collection, 64 gymnasts dropped out, leaving 410 (78%) who completed the full season data collection. Drop‐out reasons included pregnancy (*n* = 1), quitting gymnastics (*n* = 38), transitioning to high school (*n* = 6), loss of contact for over 4 weeks (*n* = 5), personal withdrawal (*n* = 5), and other reasons, such as injuries and mental health issues (*n* = 9).

Sixteen registered injuries could not be categorized regarding time‐loss because they occurred during weeks with multiple injuries for the same gymnast, making it unclear which injury the text‐message responses referred to. Additionally, 15% of the recorded injuries occurred bilaterally in the same region, with gymnasts unable to determine which side was more painful. Bilateral injuries were most commonly reported in the lower leg (33% of cases), the knee (15%), and the foot (12%).

Of the 474 gymnasts included in the study, the mean age was 14.2 years (SD 3.2). This included 128 males (mean age 15.2, SD 3.5) and 346 females (mean age 13.9, SD 3.0). Additional demographic data are presented in Table 2.

**TABLE 2:** Demographic characteristics of the gymnasts and gymnastic exposure.

Demographics	Overall group	Male	Female
	*n* = 474	*n* = 128	*n* = 346
Age, mean (SD), year	14.2 (3.2)	15.2 (3.5)	13.9 (3.0)
Body mass index, mean (SD), kg/m^2^	19.9 (2.7)	20.4 (2.6)	19.7 (2.8)
Weight, mean (SD), kg	50.2 (12.3)	56.9 (14.5)	47.8 (10.4)
Height, mean (SD), cm	162.4 (10.1)	168.4 (12.6)	160.2 (7.9)
Preferred take‐off leg^a^			
Left, *n* (%)	218 (46.0)	68 (53.1)	150 (43.4)
Right, *n* (%)	256 (54.0)	60 (46.9)	196 (56.7)
Years of gymnastic experience, mean (SD), years	5.8 (3.4)	5.7 (3.4)	5.8 (3.4)
Team level^b^			
Elite, *n* (%)	245 (51.7)	87 (68.0)	158 (45.7)
Sub‐elite, *n* (%)	217 (45.8)	35 (27.3)	182 (52.6)
Classified as having GJH (cut‐point 2/5), *n* (%)	316 (66.8)	47 (36.8)	269 (78.0)
Exposure			
Total training and competition exposure per week, mean (SD), h	6.0 (3.5)	5.4 (3.4)	6.2 (3.5)

Abbreviations: CI, confidence interval; GJH, generalized joint hypermobility; SD, standard deviation.

^a^ The leg used before entering the trampette with both legs.

^b^ 12 participants (6 male and 6 female) (2.5%) dropped out before the level of competition was recorded.

### 3.1 Response Rates

The response rates for the text‐messaging survey were 94.2%, SD 11.7 (Q1), 91.6%, SD 15.2 (Q2), 88.6%, SD 18.6 (Q3), and 86.4%, SD 20.6 (Q4), with an average response rate of 90.2%, SD 15.9. A total of 1532 injury/complaint registrations were recorded (Q1 = 1), with follow‐up telephone calls completed for 1491 cases, resulting in a classification rate of 97.3%. Of these, 109 injuries were unrelated to gymnastics, leaving 1382 gymnastic‐related injuries.

### 3.2 Overall Injury Incidence and Illness

The injuries occurred over 93 879 h of gymnastic exposure, resulting in an overall injury incidence rate of 14.7 per 1000 h. Time‐loss injuries accounted for 330 cases, including 149 acute and 181 overuse injuries, with incidence rates of 3.3, 1.5, and 1.8 per 1000 h, respectively. Both time‐loss and non‐time‐loss injuries were predominantly caused by overuse. Re‐injuries comprised 41% of all recorded injuries (569 cases). During the data collection period, total absence due to illness averaged 2.5 weeks (SD 2.3). For a detailed comparison of time‐loss and non‐time‐loss injuries, see Table 3. Demographic data and overall injury incidence by age group are presented in Appendix B.

**TABLE 3:** Injuries, injury incidence rates and overall illness.

Injuries	Overall group	Male	Female
	*n* = 474	*n* = 128	*n* = 346
All injuries, *n* (%)	1382 (100.0)	359 (26.0)	1023 (74.0)
Time‐loss, *n* (%)	330^b^ (24.2)	93 (26.2)	237 (23.4)
Acute time‐loss, *n* (%)	149 (45.2)	45 (48.4)	104 (43.9)
Training, *n* (%)	130 (87.3)	37 (82.2)	93 (89.4)
Competition, *n* (%)	19 (12.8)	8 (17.8)	11 (10.6)
Overuse time‐loss, *n* (%)	181 (54.9)	48 (51.6)	133 (56.1)
Non‐time‐loss, *n* (%)	1036^b^ (75.8)	262 (73.8)	774 (76.6)
Acute non‐time‐loss, *n* (%)	367 (35.4)	109 (41.6)	258 (33.3)
Training, *n* (%)	335 (91.3)	92 (84.4)	243 (94.2)
Competition, *n* (%)	32 (8.7)	17 (15.6)	15 (5.8)
Overuse non‐time‐loss, *n* (%)	669 (64.6)	153 (58.4)	516 (66.7)
Overall recurrent injuries, *n* (%)	569 (41.3)^c^	129 (35.9)^d^	440 (43.0)^e^
Sought medical attention, *n* (%)	453 (32.8)	110 (30.1)	343 (33.5)

Injury incidence rates^a^			
All injuries, incidence rate (95% CI)	14.7 (13.7–15.9)	17.2 (15.1–19.5)	14.0 (12.8–15.4)
Non‐time‐loss, incidence rate (95% CI)	11.4 (10.5–12.5)	13.1 (11.3–15.2)	11.0 (9.8–12.2)
Acute, incidence rate (95% CI)	4.1 (3.6–4.6)	5.4 (4.3–6.6)	3.7 (3.2–4.3)
Overuse, incidence rate (95% CI)	7.4 (6.6–8.2)	7.7 (6.3–9.4)	7.3 (6.4–8.2)
Time‐loss, incidence rate (95% CI)	3.3 (2.9–3.7)	4.1 (3.2–5.2)	3.1 (2.7–3.6)
Acute, incidence rate (95% CI)	1.5 (1.3–1.8)	2.1 (1.5–2.8)	1.3 (1.1–1.6)
Overuse, incidence rate (95% CI)	1.8 (1.5–2.1)	2.0 (1.4–2.8)	1.7 (1.4–2.1)
Sought medical attention, incidence rate (95% CI)	4.8 (4.3–5.4)	5.2 (4.1–6.6)	4.7 (4.1–5.4)

Illness			
Total absence due to illness, mean (SD), weeks	2.5 (2.3)	1.7 (1.7)	2.8 (2.4)

*Note:* Sub‐categories are calculated based on the nearest main category.

Abbreviations: CI, confidence interval; SD, standard deviation.

^a^ Incidence rate is the number of injuries per 1000 h of exposure.

^b^ Sixteen injuries were unclassifiable as non‐time‐loss or time‐loss.

^c^ Five injuries could not be categorized.

^d^ One injury could not be categorized.

^e^ Four injuries could not be categorized.

### 3.3 Pain, Restrictions and Time‐Loss

**FIGURE 2:**
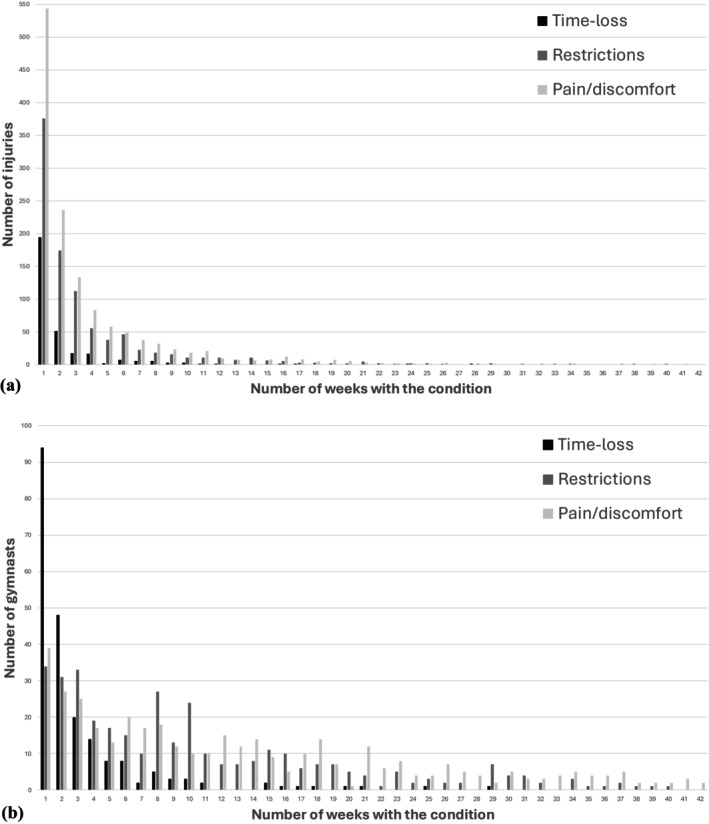
Distribution of the number of weeks with reported pain/discomfort, training restrictions, and time‐loss due to injury. (a) Number of weeks each individual injury contributed to pain/discomfort, restrictions, or time‐loss during the season. (b) Number of weeks each individual gymnast experienced pain/discomfort, restrictions, or time‐loss due to injury over the course of the season.

Of the gymnasts included in the study, 83 (18%) did not report any pain or discomfort, 127 (27%) experienced no training restrictions, and 258 (54%) did not report any time‐loss due to injury during the season. The distribution of pain/discomfort, restrictions, and time‐loss across the season—by individual gymnast and by injury—is presented in Figure 2.

### 3.4 Injury Incidence Rates by Body Region

Overall injury incidence rates by body region, including rates for acute and overuse injuries, are presented in Table 4. The body regions most affected by time‐loss injuries (per 1000 h of exposure) were the foot 0.9 (95% CI: 0.7–1.1), knee 0.6 (95% CI: 0.5–0.8), lower back 0.4 (95% CI: 0.3–0.5) and lower leg 0.3 (95% CI: 0.2–0.5) (Table 4). The incidence rate for severe time‐loss injuries was 0.5 (95% CI: 0.4–0.7).

**TABLE 4:** Incidence rates by body region.

Injured region	Incidence rate^a^ (overall) (95% CI)	Incidence rate^a^ (time‐loss) (95% CI)	Incidence rate^a^ (non‐time‐loss) (95% CI)
Foot	3.6 (3.2–4.0)	0.9 (0.7–1.1)	2.7 (2.4–3.0)
Acute	2.2 (1.9–2.5)	0.7 (0.5–0.9)	1.5 (1.3–1.8)
Overuse	1.4 (1.2–1.7)	0.2 (0.1–0.3)	1.2 (1.0–1.4)
Heel	0.9 (0.7–1.1)	0.2 (0.1–0.3)	0.7 (0.5–0.8)
Acute	0.2 (0.1–0.3)	0.04 (0.02–0.1)	0.1 (0.1–0.2)
Overuse	0.7 (0.5–0.9)	0.2 (0.1–0.3)	0.5 (0.4–0.7)
Lower leg	1.5 (1.3–1.8)	0.3 (0.2–0.5)	1.2 (1.0–1.4)
Acute	0.2 (0.2–0.4)	0.1 (0.04–0.2)	0.2 (0.1–0.3)
Overuse	1.2 (1.1–1.5)	0.3 (0.2–0.4)	1.0 (0.8–1.2)
Knee	3.2 (2.9–3.6)	0.6 (0.5–0.8)	2.6 (2.3–2.9)
Acute	0.7 (0.6–0.9)	0.2 (0.1–0.3)	0.5 (0.4–0.7)
Overuse	2.5 (2.2–2.9)	0.5 (0.3–0.6)	2.0 (1.8–2.4)
Thigh	0.7 (0.6–0.9)	0.2 (0.1–0.3)	0.6 (0.4–0.7)
Acute	0.3 (0.2–0.4)	0.1 (0.1–0.2)	0.2 (0.1–0.3)
Overuse	0.4 (0.3–0.6)	0.09 (0.04–0.2)	0.4 (0.3–0.5)
Hip and groin	1.0 (0.8–1.2)	0.1 (0.1–0.3)	0.8 (0.7–1.0)
Acute	0.3 (0.2–0.4)	0.03 (0.01–0.1)	0.2 (0.1–0.3)
Overuse	0.7 (0.6–0.9)	0.1 (0.1–0.2)	0.6 (0.5–0.8)
Buttock	0.1 (0.1–0.2)	0.01 (0.002–0.1)	0.1 (0.05–0.2)
Acute	0.04 (0.02–0.1)	0.01 (0.002–0.1)	0.03 (0.01–0.1)
Overuse	0.1 (0.04–0.2)	0 (0–0)	0.1 (0.03–0.1)
Lower back	1.5 (1.3–1.8)	0.4 (0.3–0.5)	1.1 (0.9–1.3)
Acute	0.6 (0.5–0.8)	0.1 (0.06–0.2)	0.5 (0.3–0.6)
Overuse	0.9 (0.7–1.1)	0.3 (0.2–0.4)	0.6 (0.5–0.8)
Neck	0.4 (0.2–0.5)	0.1 (0.06–0.2)	0.2 (0.2–0.4)
Acute	0.3 (0.2–0.4)	0.09 (0.04–0.2)	0.2 (0.1–0.3)
Overuse	0.1 (0.04–0.2)	0.02 (0.01–0.1)	0.05 (0.02–0.1)
Abdomen	0.1 (0.05–0.2)	0.01. (0.002–0.1)	0.1 (0.4–0.2)
Acute	0.03 (0.01–0.1)	0.01. (0.002–0.1)	0.02 (0.01–0.1)
Overuse	0.1 (0.03–0.1)	0 (0–0)	0.1 (0.03–0.1)
Chest	0.01 (0.002–0.08)	0.01 (0.002–0.1)	0 (0–0)
Acute	0.01 (0.002–0.08)	0.01 (0.002–0.1)	0 (0–0)
Overuse	0 (0–0)	0 (0–0)	0 (0–0)
Head and face	0.1 (0.05–0.2)	0.02 (0.005–0.1)	0.1 (0.03–0.1)
Acute	0.1 (0.05–0.2)	0.02 (0.005–0.1)	0.1 (0.03–0.1)
Overuse	0 (0–0)	0 (0–0)	0 (0–0)
Shoulder	0.3 (0.2–0.4)	0.1 (0.05–0.2)	0.2 (0.1–0.3)
Acute	0.1 (0.1–0.2)	0.03 (0.01–0.1)	0.1 (0.04–0.2)
Overuse	0.1 (0.1–0.3)	0.06 (0.03–0.1)	0.1 (0.04–0.2)
Elbow	0.2 (0.1–0.3)	0.04 (0.02–0.1)	0.1 (0.1–0.2)
Acute	0.1 (0.05–0.2)	0.02 (0.005–0.1)	0.1 (0.04–0.2)
Overuse	0.1 (0.04–0.2)	0.02 (0.005–0.1)	0.1 (0.03–0.1)
Forearm	0.04 (0.2–0.1)	0 (0–0)	0.04 (0.02–0.1)
Acute	0.03 (0.01–0.1)	0 (0–0)	0.03 (0.01–0.1)
Overuse	0.01 (0.002–0.1)	0 (0–0)	0.01 (0.002–0.1)
Wrist	0.9 (0.7–1.1)	0.1 (0.09–0.3)	0.7 (0.6–1.0)
Acute	0.2 (0.1–0.3)	0.04 (0.02–0.11)	0.2 (0.1–0.3)
Overuse	0.6 (0.5–0.8)	0.1 (0.06–0.2)	0.5 (0.4–0.7)
Hand	0.2 (0.1–0.3)	0.02 (0.01–0.1)	0.1 (0.1–0.3)
Acute	0.1 (0.1–0.2)	0.02 (0.01–0.1)	0.1 (0.1–0.2)
Overuse	0.03 (0.01–0.1)	0 (0–0)	0.03 (0.01–0.1)
Total	14.7 (13.7–15.9)	3.3 (2.9–3.7)	11.4 (10.5–12.5)
Acute	5.6 (5.0–6.1)	1.5 (1.3–1.8)	4.1 (3.6–4.6)
Overuse	9.1 (8.3–10.0)	1.8 (1.5–2.1)	7.4 (6.6–8.2)

Abbreviation: CI, confidence interval.

^a^ Incidence rate is the number of injuries per 1000 h of exposure.

### 3.5 Injury Distribution by Body Region

Most injuries occurred in the lower extremities, with 24.5% in the foot, 22.0% in the knee, and 10.4% each in the lower leg and lower back region. Except for the foot region, injuries in the most affected regions were primarily caused by overuse (see Table 5). Regions where time‐loss injuries most frequently led to a severe injury were the lower back (35.9%), head/face (33.3%) and wrist (29.4%) (Table 5).

**TABLE 5:** Proportion of injuries by body region and injury type and severity of time‐loss injuries.

Injured region	Total injuries/complaints	Percentage of total injuries/complaints (95% CI)	No. of time‐loss injuries	Percentage of all time‐loss injuries (95% CI)	Severe^a^ time‐loss injuriesn (%)	No. of non‐time‐loss injuries	Percentage of all non‐time‐loss injuries (95% CI)
Foot	338	24.5 (22.3–26.8)	88	26.7 (22.2–31.7)	16/88 (18.2)	247	23.8 (21.3–26.5)
Acute	207	15.0 (13.2–17.0)	68	20.6 (16.6–25.3)	9/68 (13.2)	139	13.4 (11.5–15.6)
Overuse	131	9.5 (8.0–11.1)	20	6.1 (3.9–9.2)	7/20 (35.0)	108	10.4 (8.7–12.4)
Heel	85	6.2 (5.0–7.6)	23	7.0 (4.7–10.3)	6/23 (26.1)	59	5.7 (4.4–7.3)
Acute	18	1.3 (0.8–2.1)	4	1.2 (0.5–3.2)	2/4 (50.0)	13	1.3 (0.7–2.2)
Overuse	67	4.9 (3.8–6.1)	19	5.8 (3.7–8.9)	4/19 (21.1)	46	4.4 (3.3–5.9)
Lower leg	144	10.4 (8.9–12.2)	34	10.3 (7.5–14.1)	6/34 (17.6)	108	10.4 (8.7–12.4)
Acute	23	1.7 (1.1–2.5)	7	2.1 (1.0–4.4)	3/7 (42.9)	16	1.5 (1.0–2.5)
Overuse	121	8.8 (7.4–10.4)	27	8.2 (5.7–11.7)	3/27 (11.1)	92	8.9 (7.3–10.8)
Knee	304	22.0 (19.9–24.3)	65	19.7 (15.7–24.4)	13/65 (20.0)	237	22.9 (20.4–25.5)
Acute	67	4.9 (3.8–6.1)	18	5.5 (3.5–8.5)	7/18 (38.9)	48	4.6 (3.5–6.1)
Overuse	237	17.2 (15.3–19.2)	47	14.2 (10.9–18.5)	6/47 (14.6)	189	18.2 (16.0–20.7)
Thigh	70	5.1 (4.0–6.4)	18	5.5 (3.5–8.5)	4/18 (22.2)	52	5.0 (3.8–6.5)
Acute	28	2.0 (1.4–2.9)	10	3.0 (1.6–5.6)	1/10 (10.0)	18	1.7 (1.1–2.7)
Overuse	42	3.0 (2.3–4.1)	8	2.4 (1.2–4.8)	3/8 (37.5)	34	3.3 (2.4–4.6)
Hip and groin	93	6.7 (5.5–8.2)	15	4.6 (2.8–7.4)	1/15 (6.6)	77	7.4 (6.0–9.2)
Acute	24	1.7 (1.2–2.6)	4	1.2 (0.5–3.2)	1/4 (25.0)	19	1.8 (1.2–2.9)
Overuse	69	5.0 (4.0–6.3)	11	3.3 (1.9–5.9)	0/11 (0)	58	5.6 (4.4–7.2)
Buttock	11	0.8 (0.4–1.4)	1	0.3 (0.04–2.1)	0/1 (0)	9	0.9 (0.5–1.7)
Acute	4	0.3 (0.1–0.8)	1	0.3 (0.04–2.1)	0/1 (0)	3	0.3 (0.1–0.9)
Overuse	7	0.5 (0.2–1.1)	0	0 (0.0–0.0)	N/A	6	0.6 (0.3–1.3)
Lower back	143	10.4 (8.9–12.1)	39	11.8 (8.7–15.8)	14/39 (35.9)	101	9.8 (8.1–11.7)
Acute	57	4.1 (3.2–5.3)	12	3.6 (2.1–6.3)	3/12 (25.0)	43	4.2 (3.1–5.6)
Overuse	86	6.2 (5.1–7.6)	27	8.2 (5.7–11.7)	11/27 (40.7)	58	5.6 (4.4–7.2)
Neck	33	2.4 (1.7–3.3)	10	3.0 (1.6–5.6)	1/10 (10.0)	23	2.2 (1.5–3.3)
Acute	26	1.9 (1.3–2.8)	8	2.4 (1.2–4.8)	1/8 (12.5)	18	1.7 (1.1–2.7)
Overuse	7	0.5 (0.2–1.1)	2	0.6 (0.2–2.4)	0/2 (0)	5	0.5 (0.2–1.2)
Abdomen	9	0.7 (0.3–1.3)	1	0.3 (0.04–2.1)	0/1 (0)	8	0.8 (0.4–1.5)
Acute	3	0.2 (0.1–0.7)	1	0.3 (0.04–2.1)	0/1 (0)	2	0.2 (0.1–0.8)
Overuse	6	0.4 (0.2–1.0)	0	0 (0–0)	N/A	6	0.6 (0.3–1.3)
Chest	1	0.1 (0.01–0.5)	1	0.3 (0.04–2.1)	0/1 (0)	0	0 (0–0)
Acute	1	0.1 (0.01–0.5)	1	0.3 (0.04–2.1)	0/1 (0)	0	0 (0–0)
Overuse	0	0 (0–0)	0	0 (0–0)	N/A	0	0 (0–0)
Head and face	9	0.7 (0.3–1.3)	3	0.9 (0.3–2.8)	1/3 (33.3)	5	0.5 (0.2–1.2)
Acute	9	0.7 (0.3–1.3)	3	0.9 (0.3–2.8)	1/3 (33.3)	5	0.5 (0.2–1.2)
Overuse	0	0 (0–0)	0	0 (0–0)	N/A	0	0 (0–0)
Shoulder	24	1.7 (1.2–2.6)	9	2.7 (1.4–5.2)	0/9 (0)	15	1.5 (0.9–2.4)
Acute	10	0.7 (0.4–1.3)	3	0.9 (0.3–2.8)	0/3 (0)	7	0.7 (0.3–1.4)
Overuse	14	1.0 (0.6–1.7)	6	1.8 (0.8–4.0)	0/6 (0)	8	0.8 (0.4–1.5)
Elbow	17	1.2 (0.8–2.0)	4	1.2 (0.5–3.2)	1/4 (25.0)	13	1.3 (0.7–2.2)
Acute	9	0.7 (0.3–1.3)	2	0.6 (0.2–2.4)	1/2 (50.0)	7	0.7 (0.3–1.4)
Overuse	8	0.6 (0.3–1.2)	2	0.6 (0.2–2.4)	0/2 (0)	6	0.6 (0.3–1.3)
Forearm	4	0.2 (0.1–0.8)	0	0 (0–0)	N/A	4	0.4 (0.1–1.0)
Acute	3	0.2 (0.1–0.7)	0	0 (0–0)	N/A	3	0.3 (0.1–0.9)
Overuse	1	0.1 (0.01–0.5)	0	0 (0–0)	N/A	1	0.1 (0.01–0.7)
Wrist	81	5.9 (4.7–7.2)	17	5.1 (3.2–8.1)	5/17 (29.4)	64	6.2 (4.9–7.8)
Acute	20	1.5 (0.9–2.2)	5	1.5 (0.6–3.6)	2/5 (40.0)	15	1.5 (0.9–2.4)
Overuse	61	4.4 (3.5–5.6)	12	3.6 (2.1–6.3)	3/12 (25.0)	49	4.7 (3.6–6.2)
Hand	16	1.2 (0.7–1.9)	2	0.6 (0.2–2.4)	0/2 (0)	14	1.4 (0.8–2.3)
Acute	13	0.9 (0.6–1.6)	2	0.6 (0.2–2.4)	0/2 (0)	11	1.1 (0.6–1.9)
Overuse	3	0.2 (0.1–0.7)	0	0 (0–0)	N/A	3	0.3 (0.1–0.9)
Total	1382	100	330^b^	100	68/330 (20.6)	1036^b^	100
Acute	522	37.8 (35.3–40.4)	149	45.2 (39.8–50.6)	31/149 (20.8)	367	35.4 (32.6–38.4)
Overuse	860	62.2 (59.6–64.8)	181	54.9 (49.4–60.2)	37/181 (20.4)	669	64.6 (61.6–67.4)

Abbreviation: CI, confidence interval.

^a^ Injuries including ≥ 4 time‐loss weeks from gymnastics were classified as severe.

^b^ Sixteen injuries were unclassifiable as non‐time‐loss or time‐loss.

## 4 Discussion

This is the first prospective study to closely monitor a large cohort of TeamGym gymnasts over a full season, investigating injury incidence rates for both time‐loss and non‐time‐loss injuries, along with body regional distribution, type, and severity of injuries. Data from 474 gymnasts showed an overall injury incidence rate of 14.7 per 1000 h of exposure, with three‐quarters of injuries not leading to time‐loss. Over half of the injuries affected the feet, knees, and lower back, with nearly two‐thirds caused by overuse.

### 4.1 Injury Incidence

Our findings showed an estimated injury incidence of 14.7 per 1000 h of gymnastics exposure (95% CI: 13.7–15.9), including both non‐time‐loss and time‐loss injuries. When considering only time‐loss injuries—defined as partial or total absence from training or competition during a given week due to injury—our study found a rate of 3.3 per 1000 h (95% CI: 2.9–3.7). By comparison, a previous study, using medical staff‐based injury reporting, reported a lower rate of 2.2 time‐loss injuries per 1000 h (95% CI: 1.6–2.9) [3]. The higher time‐loss rate in our study is most likely due to the use of weekly text messaging for injury registration, which previously has been shown to capture approximately 50% more injuries than traditional medical staff‐based reporting [16, 17]. Differences between our study and the study by Harringe et al. [3] may also reflect age‐related factors, as adolescent athletes are more prone to growth‐related injuries [18, 19, 20]. This could contribute to the higher incidence rates in our study, which included younger athletes than previously reported. Additionally, developments in the sport over time—such as increased difficulty and faster movements—may also have played a role in the higher injury incidence seen in our cohort.

A large difference was identified between the incidence rates of non‐time‐loss injuries (11.4 per 1000 h; 95% CI: 10.5–12.5) and time‐loss injuries (3.3 per 1000 h; 95% CI: 2.9–3.7). No previous TeamGym studies have investigated this topic and distinction.

Furthermore, 41.3% of the recorded injuries were re‐injuries. This is twice as many as previously reported in TeamGym [3] and higher than in artistic gymnastics [4]. This discrepancy is again likely due to our data collection method, which more effectively captures injuries from gymnasts [17, 21, 22]. However, the high proportion of re‐injuries emphasizes the importance of better injury management and a focus on safer return to sport.

### 4.2 Pain and Restrictions

The distribution of symptom duration among gymnasts showed that while time‐loss was generally short, many gymnasts continued to experience pain and training restrictions for considerably longer periods. These findings underscore the important challenges faced by TeamGym and other gymnastic disciplines regarding pain prevalence during gymnastic activity [23, 24]. They also raise concerns about gymnasts' ability to manage training while experiencing pain or injury.

A cross‐sectional study by Harringe et al. reported that 58% of TeamGym gymnasts compete despite injury‐related pain [6]. This, along with our findings that many gymnasts continue training with pain and restrictions during ongoing injuries, corresponds with the high non‐time‐loss injury rate observed in our study. However, how gymnasts navigate training with pain and restrictions remains unclear. Whether this practice prolongs injuries or benefits the gymnast's mental health and recovery is also unknown, emphasizing the need for further research. A 52‐week follow‐up could provide additional insights, particularly during the summer break when gymnasts typically reduce or pause training. This may offer valuable understanding of gymnast behavior during periods of lower training expectations.

### 4.3 Injury Pattern

No previous studies on TeamGym have calculated incidence rates of overuse and acute injuries separately, nor have they reported incidence rates by body region, making direct comparisons of incidence levels impossible. In this study, 64.6% of non‐time‐loss and 54.9% of time‐loss injuries were due to overuse, which is substantially larger than previously reported proportions of 21% for overuse injuries in TeamGym [3]. This discrepancy likely reflects enhanced detection of overuse injuries in our study due to weekly text messaging [21, 22] and inclusion of non‐time‐loss injuries, in contrast to the previous study reporting overuse injuries in TeamGym [3].

The most affected regions for time‐loss injuries were the foot (26.7%), knee (19.7%), and lower back (11.8%), aligning with previous TeamGym studies [3, 6, 7, 25] and comparable gymnastic disciplines [18, 26, 27, 28, 29]. However, injuries to the lower back (35.9%), head/face (33.3%), and wrist (29.4) most frequently resulted in a severe time‐loss injury. High injury prevalence in the lower back has been reported in TeamGym [3, 6, 7], and gymnasts in artistic and rhythmic gymnastics are also prone to back problems [5, 27, 28, 30]. Nevertheless, severity and duration are rarely reported and have not been explored in TeamGym until now.

In our study, upper extremity injuries accounted for a smaller portion of total injuries, but wrist injuries were the most common. Knowledge of wrist injuries in TeamGym is limited, with existing data primarily derived from an older longitudinal cohort study [25], and two studies on wrist pain prevalence [6, 24]. Wrist injuries constitute a significant proportion of upper extremity injuries in TeamGym, aligning with our findings. Similar challenges are observed in artistic gymnastics [31], where no established prevention strategies exist. In contrast, wrist injuries are rarely reported in rhythmic gymnastics [27], likely due to differences in movement demands and load distribution, highlighting a distinct difference in injury patterns between rhythmic gymnastics and TeamGym. Implementing targeted prevention strategies more rigorously in TeamGym training may help address injuries caused by repetitive wrist load, and proper technique and strengthening of wrist and core muscles have been suggested as beneficial [31].

### 4.4 Strength and Limitations

This study is the first and largest in gymnastics to collect injury and exposure data through weekly text messaging, a method available for over a decade. This approach is a significant strength, alongside the high average response rate (91.2%) and injury classification rate (97.3%). Unlike previous studies relying on coaches/medical staff for injury tracking [3, 7], our method likely captured minor congestion and overuse injuries from gymnasts themselves more effectively, reducing underreporting [17, 21, 22]. The large sample of 474 gymnasts, drawn from diverse socioeconomic areas, further strengthens the study's external validity.

However, our study also has limitations. Missing values were not imputed when calculating average exposure, which may have resulted in slight estimation errors. Furthermore, training and competition exposure were not recorded separately, as we aimed to simplify the exposure question and minimize the weekly response burden. This approach was especially important given the young age group included in the study, for whom clear and concise questions were essential to ensure reliable responses.

Despite detailed instructions, some gymnasts may have included the day of injury in their time‐loss responses (Q2), potentially overestimating time‐loss injuries and incidence rates. Additionally, the data collection method did not allow us to assess injury severity across different regions in cases of multi‐trauma; therefore, the severity of these injuries (1%) was not included in the analysis. Furthermore, the method did not allow both injuries to be tracked when simultaneous bilateral overuse injuries occurred in the same region. As a result, they were counted as a single region only, leading to an underestimation of the total number of injuries.

Finally, when a new injury or complaint occurred during an existing injury, recording of the initial injury's duration was halted. These limitations may have led to minor underestimation of severe injuries or a misclassification of non–time‐loss injuries.

### 4.5 Perspectives

The high quantity of injuries in TeamGym is likely underestimated. To reduce injury rates, it is essential to rethink current warm‐up and training routines and develop and implement targeted injury‐prevention programs. These programs should prioritize the lower extremities, especially the feet and knees, but also address body regions such as the lower back and wrists.

In other sports, injury‐prevention programs incorporating strength and neuromuscular exercises have shown significant benefits [32, 33, 34], while core stability and functional flexibility are of importance for preventing back and wrist injuries [31, 35]. In addition, a recent study on rhythmic gymnastics suggested including load management and optimized periodization in future injury‐prevention programs [36]. Furthermore, educating coaches and gymnasts about the effects of growth spurts and general injury prevalence is crucial for understanding and adherence to injury prevention routines [37, 38]. Our findings emphasize the need for a multidisciplinary team, including researchers, gymnastic coaches, and physical trainers to address lower back injuries, focusing on their etiology [39], impact of overuse injuries, and their relation to growth spurts and maturation [20, 40]. Furthermore, the role of strength training in training sessions, as well as the timing of introducing acrobatic elements/movements that stress the lower back, may benefit from being aligned with each individual gymnast's physical capacity.

Future TeamGym studies should specifically examine how age‐specific injuries, such as growth‐related versus other types of injuries, contribute to overall injury incidence.

## 5 Conclusion

TeamGym exhibits a high injury incidence rate, with three‐quarters of injuries classified as non‐time‐loss and nearly two‐thirds reported as overuse injuries. More than half of these injuries affected the feet, knees, and lower back across both sexes, while injuries to the lower back, head/face, and wrist most often resulted in severe time‐loss.

## Author Contributions

All authors fulfill criteria for authorship. C.A.‐P., B.J.‐K., and K.T. conceived the study, while C.A.‐P., M.B.C., P.H., and K.T. designed it. C.A.‐P. collected the data. All authors contributed to the analysis or interpretation of data. C.A.‐P. wrote the initial draft, and all authors critically revised it for important intellectual content and approved the final version.

## Ethics Statement

This study was approved by the Ethics Committee of the Capital Region (H‐20024056).

## Consent

Participants provided informed consent before participating in the study.

## Conflicts of Interest

The authors declare no conflicts of interest.

## Supporting information


Appendix A.

Appendix B.

## Data Availability

The data that support the findings of this study are available from the corresponding author upon reasonable request.
