# Supplementary material for: Full‐Season Injury Epidemiology in TeamGym—A Prospective Cohort Study Involving 474 Gymnasts
Source: Scand J Med Sci Sports. 2025 Sep 14;35(9):e70135. doi: 10.1111/sms.70135 (PMC12434387; doi:10.1111/sms.70135)
Supplement: Supplementary file 1 — Appendix A. [file SMS-35-e70135-s001.pdf]

**Appendix A – Structured telephone interview**

# Opfølgende telefoninterview

**Skema til registrering af skader eller smerte/ubehag**

Uge:_______

Dato for tlf. opkald:________________________________________ Ringet antal gange:_____________

Navn:____________________________________________________ ID-nummer: _________________

Klub: ____________________________________________________ Telefonnr.:___________________

Dato for skade eller opståen af smerte/ubehag (anfør den præcise dato):______________________________

**A. Er skaden eller smerte/ubehaget opstået i forbindelse med gymnastik eller på anden måde relateret til gymnastik?**

| | |
|---|---|
| 1. Ja ☐ | 2. Nej ☐ |

**B. Er der tale om en ny skade (ny smerte/ubehag) eller havde du symptomer i forvejen?**

| | |
|---|---|
| 1. Ny skade (smerte/ubehag) ☐ | 2. Forværring af eksisterende skade ☐<br><br>Er skaden (smerten/ubehaget) registreret i forrige uge eller tidligere uger på SMS? Ja ☐ / Nej ☐ |

## Skadestype

**C. Var en enkelt akut (pludselig) hændelse årsag til skaden, eller er smerten/ubehaget opstået gradvist?**

| | |
|---|---|
| 1. Akut opstået ☐ | 2. Gradvist opstået ☐ |

## Hvis akut opstået:

**D. Hvilken type skade?**

1. Led (forstuvning/forvridning) ☐
2. Muskel/sene (forstrækning) ☐
3. Fraktur (brækket knogle) ☐
4. Kontusion/slag (fx trælår) ☐
5. Andet ☐

**E. Opstod skaden ved træning eller konkurrence?**

1. Træning ☐
2. Konkurrence ☐

**F. Hvornår i træningen/konkurrencen skete skaden?**

1. Opvarmning ☐
2. Rytme ☐
3. Banespring ☐
4. Trampolinspring ☐
5. Pegasus ☐
6. Styrketræning ☐
7. Andet ☐

**G. Hvordan skete skaden?**

1. I et afsæt ☐
2. I en landing ☐
3. Ved slag/sammenstød med redskab eller anden person ☐
4. Ved et fald ☐
5. Andet ☐

## Skadens lokalitet

**H. I hvilken kropsregion er din skade (smerte/ubehag)?**

| | | |
|---|---|---|
| 1. Hoved og/eller ansigt *__ ☐ | 7. Bryst * __________ ☐ | 13. Lår __________ ☐ |
| 2. Skulder __________ ☐ | 8. Mave * __________ ☐ | 14. Knæ __________ ☐ |
| 3. Albue __________ ☐ | 9. Nakke * __________ ☐ | 15. Underben _______ ☐ |
| 4. Underarm __________ ☐ | 10. Ryg (THx, Lx, haleben) * ___ ☐ | 16. Hæl __________ ☐ |
| 5. Håndled __________ ☐ | 11. Balle __________ ☐ | 17. Fod __________ ☐ |
| 6. Hånd __________ ☐ | 12. Hofte og/eller lyske _______ ☐ | |

* Skader i disse regioner skal registreres under 3. N/A i spørgsmål I.

**I. I hvilken side sidder skaden?**

| | | |
|---|---|---|
| 1. Højre ☐ | 2. Venstre ☐ | 3. N/A ☐ |

## Omfang af fravær

**J. Har du været fraværende eller følt dig begrænset i din træning/konkurrence pga. skaden?**

| 1. Fuldt fravær ☐ | 2. Begrænset uden fravær ☐ | 3. Intet fravær ☐ |
|---|---|---|
| Du har i én eller flere dage i den kalenderuge*, hvor skaden skete **ikke været i stand til at træne/ konkurrere** på grund af din skade.<br><br>(Fravær på skadesdagen tæller ikke med)<br><br>(Dage, hvor der ikke er træning i kalenderugen tæller med) | Du har **ikke haft fuldt fravær** i én eller flere dage efter skaden, men har i én eller flere dage i den kalenderuge*, hvor skaden skete **følt dig begrænset i forbindelse med din træning/konkurrence** på grund af din skade.<br><br>(Fravær på skadesdagen tæller ikke med)<br><br>(Dage, hvor der ikke er træning i kalenderugen tæller med) | Du har **alle dage** efter skadesdagen i kalenderugen* kunnet træne/konkurrere normalt **uden** at føle dig begrænset på trods af din skade.<br><br>(Fravær på skadesdagen tæller ikke med)<br><br>(Dage, hvor der ikke er træning i kalenderugen tæller med) |

* En kalenderuge går fra mandag morgen til efterfølgende mandag morgen

## Recidiv

**K. Har du haft samme skade/problem før? Dvs. smerte/ubehag præcis samme sted som nu, men evt. i større eller mindre grad?**

| 1. Ny skade ☐ | 2. Reskade ☐ |
|---|---|
| **Hvis reskade:** | |
| Hvor længe siden? __________(uger)<br><br>Var det i projektperioden? Ja ☐ /Nej ☐ | 1 måned = 4 uger 3 måneder = 13 uger<br>6 måneder = 26 uger 12 måneder = 52 uger |

## Medicinsk kontakt

| L. Har der været kontakt til læge, fysioterapeut eller anden sundhedsfaglig person? | 1. Ja ☐ | 2. Nej ☐ |
|---|---|---|

**Evt. diagnose__________________________ Kontaktpersons uddannelse__________________________**
