# Supplementary material for: Full‐Season Injury Epidemiology in TeamGym—A Prospective Cohort Study Involving 474 Gymnasts
Source: Scand J Med Sci Sports. 2025 Sep 14;35(9):e70135. doi: 10.1111/sms.70135 (PMC12434387; doi:10.1111/sms.70135)
Supplement: Supplementary file 2 — Appendix B. [file SMS-35-e70135-s002.pdf]

APPENDIX B

Table 6. Demographics and Exposure by age groups.

Demographics	Age group 10-13 years (Mini)			Age group 14-16 years (Junior)			Age group 17-30 years (Senior)		
	**All** *n = 232*	**Boys** *n = 43*	**Girls** *n = 189*	**All** *n = 141*	**Boys** *n = 36*	**Girls** *n = 105*	**All** *n = 101*	**Males** *n = 49*	**Females** *n = 52*
Age, mean (SD), year	11.9 (1.0)	11.6 (1.0)	12.0 (1.0)	14.4 (0.6)	14.4 (0.6)	14.4 (0.6)	19.2 (2.6)	18.9 (2.3)	19.6 (2.9)
Body-mass Index, mean (SD) kg/m^2	18.5 (2.1)	18.1 (1.8)	18.6 (2.2)	20.3 (2.5)	20.3 (2.1)	20.2 (2.7)	22.5 (2.2)	22.5 (1.7)	22.4 (2.6)
Weight, mean (SD) kg	42.0 (8.0)	41.7 (8.8)	42.1 (7.8)	52.5 (8.2)	56.2 (8.5)	51.2 (7.7)	66.0 (7.9)	70.8 (5.5)	61.5 (7.1)
Height, mean (SD), cm	156.5 (8.4)	155.1 (10.1)	156.8 (7.9)	165.5 (6.7)	171.5 (6.6)	163.4 (5.4)	171.9 (8.3)	177.7 (6.5)	166.4 (5.7)
Preferred take-off leg									
Left, n (%)	106 (45.7)	22 (51.1)	84 (44.4)	58 (41.1)	19 (52.8)	39 (37.1)	54 (53.5)	27 (55.1)	27 (51.9)
Right, n (%)	126 (54.3)	21 (48.8)	105 (55.6)	83 (58.9)	17 (47.2)	66 (62.9)	47 (46.5)	22 (44.9)	25 (48.1)
Years of gymnastic experience, mean SD, years	4.4 (2.0)	3.8 (2.2)	4.5 (1.9)	5.9 (2.3)	6.0 (2.0)	5.9 (2.4)	8.7 (4.9)	7.1 (4.2)	10.3 (4.9)
Team level									
Elite	48 (20.7)	8 (18.6)	40 (21.2)	98 (69.5)	31 (86.1)	67 (63.8)	99 (98.2)	48 (98.0)	51 (98.1)
Sub-elite	180 (77.6)	33 (76.7)	147 (77.8)	37 (26.2)	2 (5.6)	35 (33.3)	0 (0)	0 (0)	0 (0)
Classified as having GJH (cut-point 2/5), n (%)	146 (63.2)	9 (20.9)	137 (72.9)	105 (74.5)	17 (47.2)	88 (83.8)	65 (64.4)	21 (42.9)	44 (84.6)
**Exposure**									
Total training and competition exposure per week, mean (SD), hours	6.1 (3.3)	5.4 (3.1)	6.2 (3.3)	6.3 (3.7)	5.4 (2.9)	6.6 (3.9)	5.5 (3.6)	5.5 (3.9)	5.4 (3.3)

Abbreviations: GJH, generalized joint hypermobility; SD, standard deviations; CI, confidence interval.

Table 7. Injuries and Injury Incidence Rates by age groups.

	Age group 10-13 years (Mini)			Age group 14-16 years (Junior)			Age group 17-30 years (Senior)		
**Injuries**	**All** ***n = 232***	**Boys** ***n = 43***	**Girls** ***n = 189***	**All** ***n = 141***	**Boys** ***n = 36***	**Girls** ***n = 105***	**All** ***n = 101***	**Males** ***n = 49***	**Females** ***n = 52***
All injuries, n (%)	677 (100.0)	112 (16.5)	565 (83.5)	437 (100.0)	118 (27.0)	319 (73.0)	268 (100.0)	129 (48.1)	139 (51.9)
Time loss, n (%)	154 (22.7)	29 (25.9)	125 (22.1)	89 (20.4)	24 (20.3)	65 (20.4)	87 (32.5)	40 (31.0)	47 (33.8)
Acute time-loss, n (%)	67 (43.5)	13 (44.8)	54 (43.2)	40 (44.9)	8 (33.3)	32 (49.2)	42 (48.3)	24 (60.0)	18 (38.3)
Training, n (%)	63 (94.0)	12 (92.3)	51 (94.4)	37 (92.5)	8 (100.0)	29 (90.6)	30 (71.4)	17 (70.8)	13 (72.2)
Competition, n (%)	4 (6.0)	1 (7.7)	3 (5.6)	3 (7.5)	0 (0)	3 (9.4)	12 (28.6)	7 (29.2)	5 (27.8)
Overuse time-loss, n (%)	87 (56.5)	16 (55.2)	71 (56.8)	49 (55.1)	16 (66.7)	33 (50.8)	45 (5151.7)	16 (40.0)	29 (61.7)
Non-time-loss, n (%)	513 (75.8)	83 (74.1)	430 (76.1)	342 (78.3)	90 (76.3)	252 (79.0)	181 (67.5)	89 (69.0)	92 (66.2)
Acute non-time-loss, n (%)	169 (32.9)	32 (38.6)	137 (31.9)	125 (36.5)	32 (35.6)	93 (36.9)	73 (40.3)	45 (50.6)	28 (30.4)
Training, n (%)	162 (95.9)	29 (90.6)	133 (97.1)	112 (89.6)	27 (84.4)	85 (91.4)	61 (83.6)	36 (80.0)	25 (89.3)
Competition, n (%)	7 (4.1)	3 (9.4)	4 (2.9)	13 10.4)	5 (15.6)	8 (8.6)	12 (16.4)	9 (20.0)	3 (10.7)
Overuse non-time-loss, n (%)	344 (67.1)	51 (61.5)	293 (68.1)	217 (63.5)	58 (64.4)	159 (63.1)	108 (59.7)	44 (49.4)	64 (69.6)
Overall recurrent injuries, n (%)	276 (40.9)[1]	38 (33.9)	238 (42.1)	176 (40.4)[2]	44 (37.3)	132 (41.4)	117 (44.0)[1]	47 (36,4)	70 (50.4)
Sought medical attention, n (%)	207 (30.6)	30 (26.8)	177 (31.3)	157 (36.0)	36 (30.5)	121 (37.9)	89 (33.2)	44 (34.1)	45 (32.4)
**Injury Incidence Rates**[a]									
All injuries, incidence rate (95% CI)	14.2 (12.8-15.8)	15.6 (12.5-19.4)	13.9 (12.4-15.7)	14.8 (12.8-17.1)	19.3 (15.1-24.7)	13.6 (11.5-16.1)	16.1 (13.8-18.9)	16.9 (13.7-20.8)	15.5 (12.2-19.7)
Time-loss, incidence rate (95% CI)	3.0 (2.5-3.6)	3.6 (2.3-5.7)	2.9 (2.4-3.5)	2.9 (2.2-3.7)	3.6 (2.2-5.9)	2.7 (2.0-3.6)	4.9 (3.9-6.1)	4.8 (3.5-6.7)	4.9 (3.5-6.9)
Acute, incidence rate (95% CI)	1.3 (1.0-1.7)	1.7 (0.9-3.1)	1.2 (0.9-1.6)	1.4 (1.0-1.9)	1.3 (0.2-7.6)	1.4 (1.0-1.9)	2.4 (1.8-3.3)	3.0 (2.0-4.5)	1.9 (1.2-3.0)
Overuse, incidence rate (95% CI)	1.7 (1.4-2.2)	2.0 (1.1-3.6)	1.7 (1.3-2.2)	1.5 (1.0-2.2)	2.3 (1.2-4.4)	1.3 (0.8-2.1)	2.5 (1.7-3.5)	1.8 (1.2-2.9)	3.0 (1.8-5.1)
Non-time-loss, incidence rate (95% CI)	11.2 (9.9-12.7)	12.0 (9.3-15.5)	11.0 (9.6-12.8)	11.9 (10.2-13.9)	15.7 (12.1-20.5)	10.9 (9.1-13.11)	11.3 (9.1-13.9)	12.0 (9.3-15.5)	10.6 (7.6-14.8)
Acute, incidence rate (95% CI)	3.8 (3.1-4.5)	4.6 (3.2-6.7)	3.6 (2.9-4.4)	4.3 (3.5-5.3)	5.4 (3.5-8.3)	4.0 (3.2-5.1)	4.5 (3.4-6.0)	6.0 (4.4-8.3)	3.2 (2.0-5.3)
Overuse, incidence rate (95% CI)	7.4 (6.5-8.6)	7.4 (5.4-10.0)	7.4 (6.3-8.7)	7.7 (6.3-9.3)	10.3 (7.2-14.8)	7.0 (5.6-8.7)	7.0 (5.4-8.9)	5.6 (4.0-7.8)	8.3 (5.8-11.7)

Abbreviations: CI, confidence interval. [a]Incidence rate is the number of injuries per 1,000 hours of exposure. [1]Two injuries could not be categorized, [2]One injury could not be categorized,
